# Supplementary material for: Association between oral microbiome and seven types of cancers in East Asian population: a two-sample Mendelian randomization analysis
Source: Front Mol Biosci. 2023 Nov 21;10:1327893. doi: 10.3389/fmolb.2023.1327893 (PMC10702768; doi:10.3389/fmolb.2023.1327893)
Supplement: Supplementary file 1 [file DataSheet1.ZIP › Supplementary Materials/MR plots of saliva/saliva═╝/colorectal cancer/pheno.3285_to_colorectal cancer_leave_one_out.pdf]

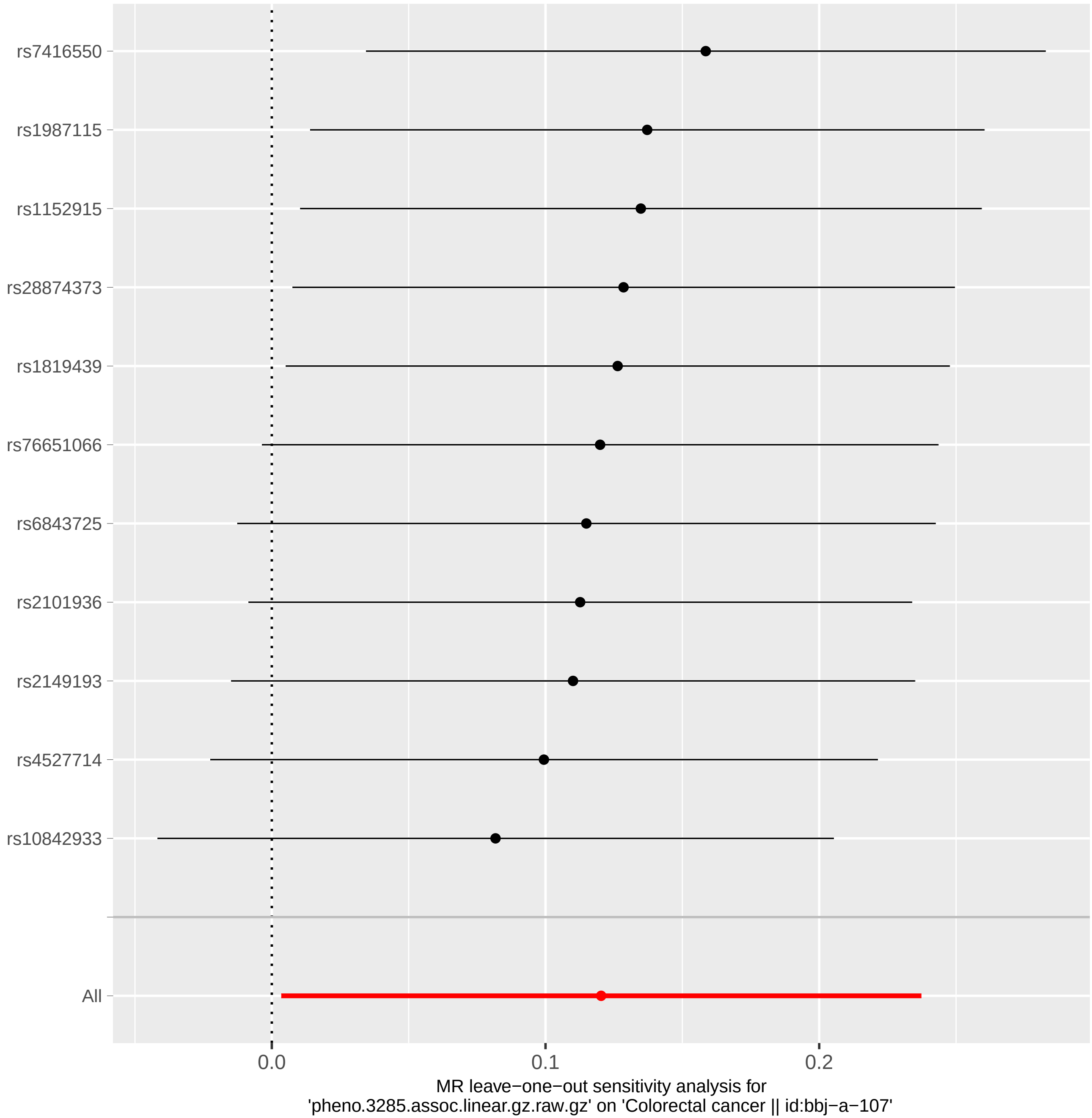
rs7416550
rs1987115
rs1152915
rs28874373
rs1819439
rs76651066
rs6843725
rs2101936
rs2149193
rs4527714
rs10842933
All
0.0
0.1
0.2
MR leave−one−out sensitivity analysis for
'pheno.3285.assoc.linear.gz.raw.gz' on 'Colorectal cancer || id:bbj−a−107'
